# Efficacy and Safety of Programmed Death-1/Programmed Death-Ligand 1 Inhibitor for Metastatic Urothelial Carcinoma: A Systematic Review and Meta-Analysis

**DOI:** 10.3390/curroncol30110722

**Published:** 2023-11-17

**Authors:** Pei-Fei Liao, Ping-Yu Wang, Tzu-Rong Peng

**Affiliations:** Department of Pharmacy, Taipei Tzu Chi Hospital, Buddhist Tzu Chi Medical Foundation, New Taipei City 23142, Taiwan

**Keywords:** checkpoint inhibitors, metastatic urothelial cancer, chemotherapy, PD-1/PD-L1 inhibitor, overall survival

## Abstract

**Objective**: The purpose of this study was to evaluate the efficacy and safety of programmed death-1 (PD-1)/programmed death-ligand 1 (PD-L1) inhibitors for the treatment of metastatic urothelial carcinoma (mUC). **Methods:** A literature search was conducted of PubMed, EMBASE, and the Cochrane Library and was limited to the English literature. Randomized controlled trials (RCTs) published up to July 2022 were considered for inclusion. The outcomes were progression-free survival (PFS), overall survival (OS), objective response rate (ORR), and grade ≥ 3 treatment-related AEs (TRAE). Subgroup analysis was performed based on the PD-L1 expression status, and the differences between first- and second-line PD-1/PD-L1 inhibitors were estimated. **Results:** We included five RCTs comprising 3584 patients in the analysis. Compared with chemotherapy alone, the use of PD-1/PD-L1 inhibitors as monotherapy did not significantly prolong OS [hazard ratios (HR), 0.90; 95% CI, 0.81–1.00] or PFS (HR, 1.12; 95% CI, 0.95–1.32). However, the PD-1/PD-L1 inhibitor combined with chemotherapy significantly improved both OS (HR, 0.85; 95% CI, 0.74–0.96) and PFS (HR, 0.80; 95% CI, 0.71–0.90). Additionally, subgroup analysis showed that in mUC with PD-L1 expression ≥ 5%, treatment with the PD-1/PD-L1 inhibitor alone did not reduce the risk of death. Safety analysis showed that the PD-1/PD-L1 inhibitor alone did not significantly increase the incidence rates of grade ≥ 3 TRAEs. **Conclusions:** The results show that use of the PD-1/PD-L1 inhibitor alone as first-line treatment is similar to chemotherapy in terms of both survival and response rates. However, the PD-1/PD-L1 inhibitor plus chemotherapy has a significant benefit in terms of PFS or OS. Nonetheless, more RCTs are warranted to evaluate efficiency and safety in the combination regimen of chemotherapy and PD-1/PD-L1 inhibitors.

## 1. Introduction

The ninth most common cancer in the world is urothelial carcinoma (UC) of the bladder, accounting for 90% of all bladder tumors that originate from the bladder or upper urinary tracts (renal pelvis and ureter) [1,2]. According to statistics from Taiwan, in 2012, the age-standardized incidence rate (ASR) of urological cancers, such as ureteral cancer, was 1.90 per 100,000 people. These rates were higher than those in many other Asian countries. Among them, the prevalence of upper urinary tract urothelial carcinoma (UTUC) was as high as 30% of all UC cases [3,4]. In Western nations, such as North America and Europe, the male-to-female ratio ranges between 2:1 and 4:1 of the normal UTUC prevalence [5,6], whereas in Taiwan, it is 1:1.3 [5,6]. Compared with the incidence in other countries, the incidence of UTUC in Taiwan is abnormally high, especially in women [7,8]. Therefore, UTUC is indeed a crucial health problem in Taiwan.

Despite the 96% five-year survival rate for carcinoma in situ of the urinary bladder, metastatic urinary bladder cancer (mUC) is an aggressive cancer associated with a dismal prognosis, with 5-year survival rates below 6% [9]. Given the chemo-sensitivity factor, platinum-based combination chemotherapy (cisplatin or carboplatin) has long been used as the standard first-line treatment for mUC [10,11]. After initiation of first-line platinum-based chemotherapy, the median overall survival (OS) is approximately 12.5–14 months [12,13]. Nevertheless, patients diagnosed with UTUC do not qualify for first-line chemotherapy due to poor performance status [14]. Despite receiving second-line therapy with paclitaxel, pemetrexed, docetaxel, and vinflunine, survival rates have been modest, with a median OS of approximately 5–8 months [15,16].

In recent years, various types of cancer have been investigated with immune checkpoint inhibitors (ICI), such as inhibitors of programmed death-ligand 1 (PD-L1) or its receptor, programmed death-1 (PD-1). Atezolizumab and pembrolizumab have been suggested as first-line treatment for patients with metastatic UC (mUC), who are ineligible for cisplatin therapy and have a high expression of PD-L1 (based on the results of a single-arm phase II trial) [17,18,19,20]. Additionally, combinations of PD-1/PD-L1 inhibitors with other immunotherapies and targeted agents are currently being studied to improve outcomes in all stages of UC. Therefore, the purpose of this meta-analysis was to study the effectiveness and safety of PD-1/PD-L1 inhibitors in the treatment of mUC. Subgroup analyses of OS and PFS were also based on the sequence of treatment (first- and second-line treatment), PD-L1 expression level, and study methodologies (PD-1/PD-L1 inhibitor plus chemotherapy versus chemotherapy alone).

## 2. Methods

### 2.1. Literature Search Strategy 

This systematic review and meta-analysis was performed in accordance with the Preferred Reporting Items for Systematic Reviews and Meta-Analyses (PRISMA) guidelines [21,22]. We searched databases, including PubMed, EMBASE, and the Cochrane Library, for eligible studies dating from inception to July 2022. We used the following keywords: avelumab, atezoli-zumab, durvalumab, pembrolizumab, nivolumab, checkpoint inhibitor, PD-1, PD-L1, metastatic urothelial cancer, bladder cancer, tumor metastasis, and randomized controlled trial (RCT). Additionally, free terms and medical subject headings (MeSH) were used as search strategies for titles or abstracts. The literature search was limited to human studies and the English language literature.

### 2.2. Study Selection

The inclusion criteria for our study were as follows: (1) phase III RCT; (2) patients receiving anti-PD-1 or anti-PD-L-1 inhibitors for advanced urothelial cancer; (3) trials that report objective response rate (ORR), PFS, and OS. The exclusion criteria were as follows: (1) case reports, review, and conference abstracts; (2) unable to extract research-related data.

### 2.3. Data Extraction and Quality Assessment

We used a standardized format to extract data. The following information was extracted: author, year, treatment regimens, line of treatment, median PFS and OS with relative hazard ratios (HR) and 95% confidence intervals (CI), the proportion of partial or complete response, and the incidence of adverse events (AEs). The risk of bias in each study was assessed using Cochrane’s risk-of-bias tool [23]. 

### 2.4. Statistical Analysis and Data Synthesis

This study used Review Manager software (RevMan) (version 5.4; Oxford, UK) and Comprehensive Meta-Analysis (CMA) software for statistical analysis [24]. This study was conducted using the random-effects model under the assumption of significant heterogeneity [25]. The effect sizes are expressed as HR, odds ratio (OR), or risk ratio (RR) with a 95% CI. The heterogeneity among these studies was assessed using the chi-square and I-square test, which was defined as *p* < 0.1 or *I*^2^ > 50% [26,27]. 

## 3. Results

### 3.1. Study Selection

**Figure 1:**
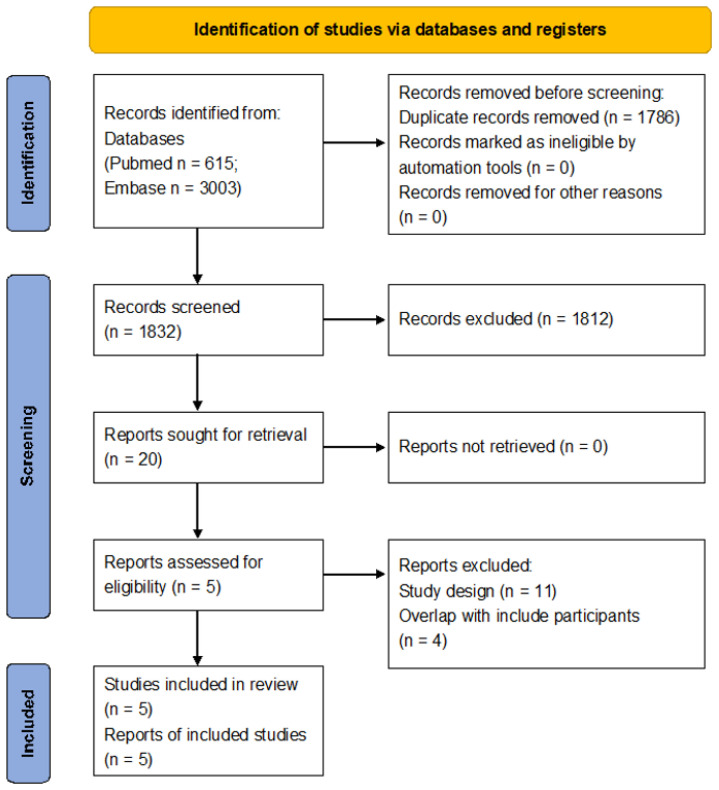
Preferred Reporting Items for Systematic Reviews and Meta-Analyses (PRISMA) 2020 flow diagram.

A total of 3618 results were found after searching the database. We eliminated 1786 entries due to duplicate material and 1812 entries due to differences in title and abstract evaluation. The final 20 papers were evaluated for full-text information, with 15 of them being discarded due to overlap in research design and subjects. Finally, five articles were included in this analysis (Figure 1) [28,29,30,31,32]. 

### 3.2. Characteristics and Risk of Bias in Included Trials

A total of 3584 patients with mUC were ultimately included in the meta-analysis, of whom 1752 received ICI and 1832 received chemotherapy alone. Of these, three studies included patients treated with anti-PD-L1 therapy [28,29,30] and two studies included patients treated with anti-PD-1 therapy [31,32]. Table 1 summarizes the baseline characteristics of these trials. All included studies were considered to have a low risk of bias because the authors explained the principles of randomization in detail. Table 2 shows the risk of bias elements for each study reviewed.

### 3.3. Efficacy

**Figure 2:**
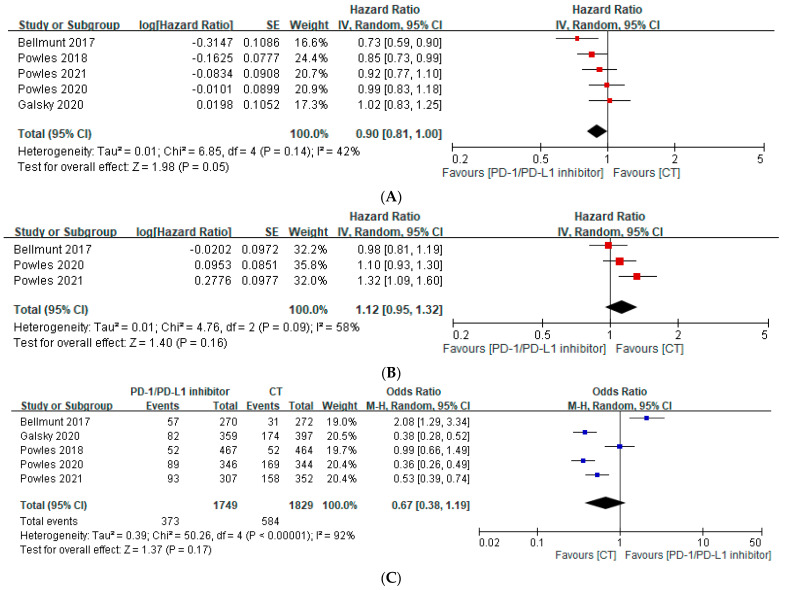
Forest plot of the efficacy of immune checkpoint inhibitors in treating patients with metastatic urothelial cancer [28,29,30,31,32]. (**A**) Pooled median overall survival rate. (**B**) Pooled median progression-free survival rate. (**C**) Pooled objective response rate. CT, chemotherapy; PD-1, programmed death-1; PFS, progression-free survival; PD-L1, programmed death-ligand 1.

This meta-analysis shows that in patients with mUC, the PD-1/PD-L1 inhibitor as monotherapy did not reduce the risk of death, compared with patients in the chemotherapy groups. The median OS of patients treated with the PD-1/PD-L1 inhibitor was not better than that of patients treated with chemotherapy alone (HR, 0.90; 95% CI, 0.81–1.00; *p* = 0.06; *I*^2^ = 42%) (Figure 2A). As shown in Figure 2B, the PD-1/PD-L1 inhibitor had the same effect on PFS as on OS (HR, 1.12; 95% CI, 0.95–1.32; *p* = 0.16; *I*^2^ = 58%). Similarly, the statistical analysis also showed no significant difference in ORR in the PD-1/PD-L1 inhibitor group compared with the chemotherapy group (OR, 0.67; 95% CI, 0.38–1.19; *p* = 0.17; *I*^2^ = 92%) (Figure 2C).

### 3.4. Treatment-Related Adverse Effects (TRAE)

Our meta-analyses revealed that the pooled RR of hematologic AE was 0.05 (95% CI, 0.02–0.10; *p* < 0.001), and the pooled RR of non-hematologic AE was 0.41 (95% CI, 0.28–0.59; *p* = 0.13). These findings implied that, compared with chemotherapy alone, PD-1/PD-L1 inhibitors did not significantly increase the incidence rates of grade ≥ 3 AE. The RR of grade ≥ 3 AE is summarized in Table 3.

### 3.5. Subgroup Analysis

To investigate the effect of each parameter on outcomes, we performed subgroup analysis according to PD-L1 expression status, study methodologies (PD-1/PD-L1 inhibitor plus chemotherapy vs. chemotherapy alone), and lines of treatment (first- and second-line). Subgroup meta-analysis using various factors is summarized in Table 4.

#### 3.5.1. Efficacy of PD-1/PD-L1 Inhibitor and PD-L1 Expression Status

**Figure 3:**
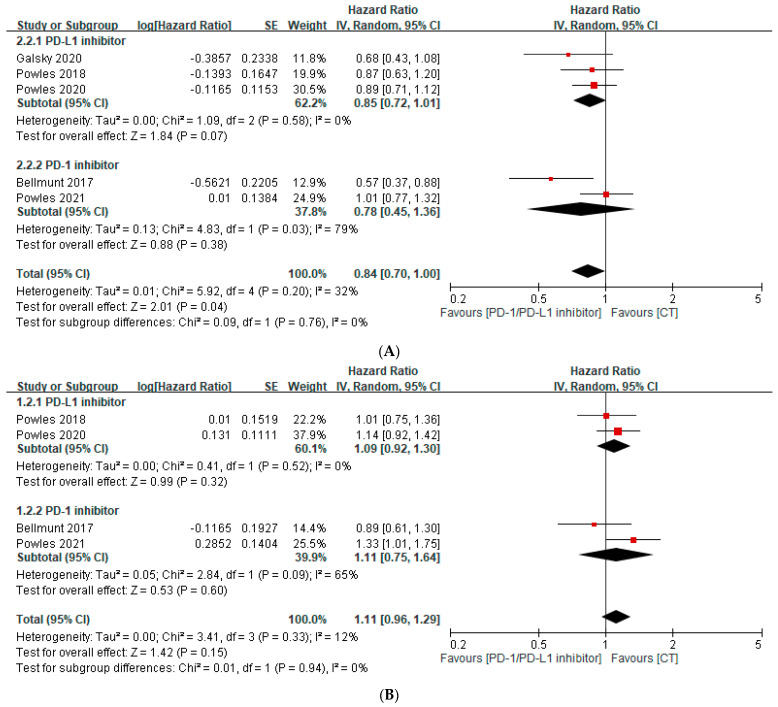

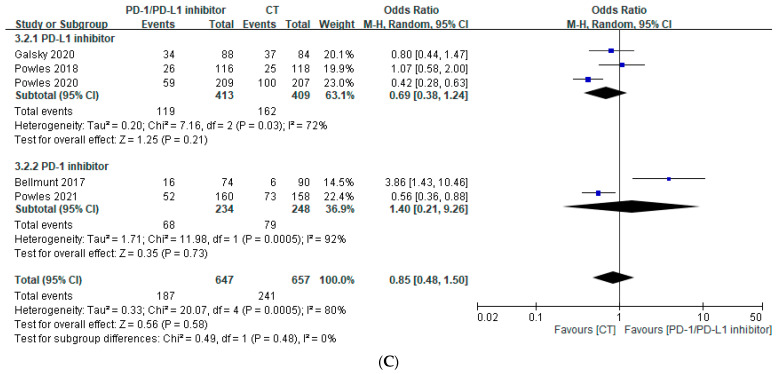
Forest plot of the efficacy of immune checkpoint inhibitors in treating patients with *PD-L1 expression ≥ 5%* (*IC2*/*3*) [28,29,30,31,32]. (**A**) Pooled median overall survival rate. (**B**) Pooled median progression-free survival rate. (**C**) Pooled objective response rate. CT, chemotherapy; PD-1, programmed death-1; PFS, progression-free survival; PD-L1, programmed death-ligand 1.

An analysis of subgroups revealed that the PD-1/PD-L1 inhibitor did not improve the rate of overall survival in patients with PD-L1-expressing immune cells on ≥5% of the tumor area (IC2/3 per immunohistochemistry assay), compared with chemotherapy alone (HR, 0.84; 95% CI, 0.70–1.00; *p* = 0.04) (Figure 3A). Similar to the OS results, within the *IC2*/*3 group*, the PFS and ORR in the PD-1/PD-L1 inhibitor subgroup were not significantly different, compared with the chemotherapy alone group (HR, 1.11; 95% CI, 0.96–1.29; *p* = 0.15) (Figure 3B) (OR, 0.85; 95% CI, 0.48–1.50; *p* = 0.58) (Figure 3C).

#### 3.5.2. Efficacy of Combined PD-1/PD-L1 Inhibitor and Chemotherapy

An analysis of subgroups revealed that patients with mUC who received the PD-1/PD-L1 inhibitor plus chemotherapy experienced significant improvement in both OS and PFS, compared with those who received chemotherapy alone (HR, 0.85; 95% CI, 0.74–0.96; *p* = 0.01) (OR, 0.80; 95% CI, 0.71–0.90; *p* < 0.001). Moreover, the PD-1/PD-L1 inhibitor plus chemotherapy subgroup had higher response rates than the chemotherapy alone group (OR, 1.30; 95% CI, 1.02–1.66; *p* = 0.03).

#### 3.5.3. Efficacy of PD-1/PD-L1 Inhibitor on Different Treatment Lines

In the subgroup analysis between first- and second-line PD-1/PD-L1 inhibitors, the differences in PFS and ORR between first-line and second-line PD-1/PD-L1 inhibitor treatment were not statistically significant. Interestingly, we found that OS in the first-line PD-1/PD-L1 inhibitor subgroup was not significantly different from that in the chemotherapy alone group (HR, 0.97; 95% CI, 0.87–1.08; *p* = 0.60). However, the second-line PD-1/PD-L1 inhibitor subgroup was statistically different from the chemotherapy alone group (HR, 0.82; 95% CI: 0.69–0.93, *p* = 0.003).

## 4. Discussion

We performed a systematic review and meta-analysis aimed at exploring the efficacy of ICI—in terms of PFS, OS, and ORR—in patients who progressed after standard chemotherapy, involving first- and second-line, and PD-1/PD-L1 inhibitor, combination therapy. In addition to an intention-to-treat (ITT) analysis in advanced or metastatic UC patients, we also focused on randomized clinical trials. When compared with chemotherapy alone, Mori et al. [33] found significant improvements in OS (HR, 0.85; 95% CI: 0.76–0.94; *p* = 0.002), PFS (HR, 0.80; 95% CI: 0.71–0.90; *p* < 0.001), and ORR (OR, 0.77; 95% CI, 0.63–0.94; *p* = 0.01) outcomes for patients with mUC treated with ICI combination therapy as a first-line treatment. Despite being consistent with our analysis, we still found a difference in the included DANUBE trial [30]. A combination therapy group in the DANUBE trial used ICI plus tremelimumab, an antibody targeting the immune checkpoint T-lymphocyte-associated protein 4 (CTLA-4), rather than conventional chemotherapy. Due to the study design, we decided not to include the data on combination therapy in the DANUBE trial, to avoid having the ICI combination therapy results amplified.

Based on the available data from the included studies, we discovered that PD-1/PD-L1 inhibitors as a second-line therapy reduced the risk of death in mUC patients (HR, 0.82; 95% CI: 0.69–0.93; *p* = 0.003), but similar outcomes were not obtained in first-line therapy. Although the conclusion of Ciccarese et al. [34] was similar to the results of our analysis (fixed-effect; HR, 0.81; 95% CI: 0.71–0.92; *p* < 0.001), the study further analyzed immunotherapy and other cytotoxic drugs (taxanes or vinflunine) and discovered that ICI outperformed taxanes (fixed-effect; HR, 0.75; 95% CI 0.64–0.87; *p* < 0.001), but not vinflunine (random-effect; HR, 0.80; 95% CI 0.55–1.17; *p* = 0.26), in terms of OS. Yoon et al. [35] showed that neither atezolizumab nor pembrolizumab delivered any significant improvement in OS in patients with mUC when compared with vinflunine in a network meta-analysis. As a result, we believe that the main explanation was a combination of data from two studies [29,31] that used different ICIs, resulting in disparities in the PD-1/PD-L1 inhibitor and cytotoxic agent findings. Furthermore, there has been no published meta-analysis that includes a phase III trial or RCTs on PD-1/PD-L1 inhibitors as second-line therapy in mUC patients.

T-cell activation is suppressed and the antitumor immune response is reduced when the PD-1 receptor binds to one of its primary ligands, PD-L1 or PD-L2. To avoid immune surveillance, many tumor cells, including mUC, overexpress PD-L1 [36]. According to the published literature, the expression of PD-L1 within tumor microenvironments is predictive of the response to ICI monotherapies in melanoma, NSCLC, and bladder cancer, but this was not true in all of the studies [37]. Notably, in certain tumor types, such as bladder cancer, the predictive value is primarily observed for PD-L1 expression on immune-infiltrating cells rather than tumor cells, whereas for NSCLC, PD-L1 expression on both immune cells and tumor cells has shown predictive value. These observations require experimental studies to dissect which of the two cell compartments is more relevant in PD-L1-mediated suppression of tumor control [37]. Furthermore, higher grades, higher stages, and poorer outcomes in UC have also been associated with PD-L1 expression in tumor cells [38]. Interestingly, another study in the literature [39] found that the predictive value for bladder cancer is primarily determined by the expression of PD-L1 on immune-infiltrating cells rather than tumor cells, while for NSCLC, both immune cells and tumor cells have shown PD-L1 expression to be predictive [40]. In addition, recent phase III trials in UC using PD-1/PD-L1 inhibitors [31] indicated that PD-L1 expression plays only a minor role in predicting a good therapeutic response. We provide aggregated data from five RCTs on the efficacy of PD-1/PD-L1 inhibitors in the ≥5% PD-L1 expression status threshold, determining whether survival rate or response rate results show no statistically significant difference in efficacy when compared with chemotherapy alone. Another meta-analysis has looked at the efficacy and/or safety of PD-(L)1-containing therapies based on the patient’s PD-L1 status [33,41,42]. 

Furthermore, we found some consistent results in real-world studies. A retrospective study by Tural et al. [43] summarized the results of first-line atezolizumab treatment in mUC. The results showed that median PFS and OS were 3.8 and 9.8 months. A prospective clinical trial by Sternberg et al. [44] in 2019 had similar results, with median PFS and OS of 2.2 and 8.7 months, respectively. In 2017, the US Food and Drug Administration (FDA) accelerated the approval of atezolizumab monotherapy for the first-line treatment of metastatic urothelial carcinoma in patients who cannot receive cisplatin. Nevertheless, according to real-world data and the results of the phase III trial IMvigor130, treatment with atezolizumab alone or in combination with platinum-based chemotherapy did not significantly improve OS [28]. As a result, atezolizumab was voluntarily withdrawn from the United States in November 2022 for patients with advanced or metastatic urothelial carcinoma, who do not qualify for chemotherapy containing cisplatin and whose tumors express PD-L1, and for those who do not qualify for chemotherapy containing platinum.

The efficacy of a PD-1/PD-L1 inhibitor as monotherapy for tumor growth suppression in metastatic areas is unknown. However, given the safety concerns, we aggregated data from the included trials, which demonstrated that patients taking chemotherapy had significantly more serious adverse effects (≥grade 3) than hematologic adverse effects (RR, 0.05; 95% CI, 0.02–0.10; *p* < 0.001). Moreover, non-hematologic adverse effects also showed similar results (RR, 0.41; 95% CI, 0.28–0.59; *p* = 0.13). PD-1/PD-L1 inhibitors may have a higher safety profile than chemotherapy. This means that, as monotherapy, PD-1/PD-L1 inhibitors may provide a viable option for patients who are ineligible for platinum-based treatment. In addition, although we did not mention pooled data in our study, many other risk factors, such as pneumonitis, were also of concern to us. In a previous meta-analysis by Su et al. [45] in 2019, the results indicated that, when compared with chemotherapy, PD-1 inhibitors were associated with significant increases in grade 1–5 (RR, 5.17; 95% CI, 2.82–9.47; *p* < 0.001) and grade 3–5 pneumonitis (RR, 4.14; 95% CI, 1.82–9.42; *p* < 0.001), but not in pneumonia. PD-L1 inhibitors showed significant increases in grade 1–5 pneumonitis (RR, 3.25; 95% CI, 1.61–6.57; *p* < 0.001) and pneumonia (RR, 2.11; 95% CI, 1.20–3.70; *p* < 0.001). A network meta-analysis by Chen et al. [46] found that conventional chemotherapy had the lowest risk of immune-related pneumonitis (IRP) grades 1–5, compared with ICI monotherapy (OR, 0.16; 95% CI, 0.09–0.25), dual ICI combination therapy (OR, 0.09; 95% CI, 0.04–0.18), and ICI combined with chemotherapy (OR, 0.34; 95% CI, 0.21–0.59). Furthermore, monotherapy with ICI (OR, 2.14; 95% CI, 1.12–4.80) and dual ICI combinations (OR, 3.86; 95% CI, 1.69–9.89) were associated with a noticeably higher rate of grade 1–5 IRP, compared with ICI plus chemotherapy. Furthermore, Sharma et al. [47] reported that, in the CheckMate 032 trial, grade 5 treatment-related pneumonitis occurred in two patients receiving nivolumab (PD-1 inhibitors) monotherapy (3 mg/kg), and nivolumab 3 mg/kg plus ipilimumab (CTLA-4 inhibitors) 1 mg/kg, respectively, compared with nivolumab 1 mg/kg plus ipilimumab 3 mg/kg. Results show that immune-related pneumonia is more common after receiving ICI treatment, and that the dose of ICI and the incidence are related. According to the DANUBE study [30], the most serious treatment-related adverse event was pneumonia in two patients in the durvalumab group and three patients in the durvalumab plus tremelimumab group, compared with the chemotherapy group. Other studies have found that combined CTLA-4 and PD-1 inhibitors could generate higher incidences of pneumonitis than either blockade individually [48,49,50]. There is no doubt that ICI can activate T cells to fight tumor cells, but those activated T cells can also attack normal tissues [51] and cause immune-related lung toxicity. Furthermore, a highly activated immune system might produce excess inflammatory cytokines (including interleukin-17) and autoantibodies [52]. CTLA-4 inhibitors regulate T-cell proliferation at an early stage in the immune response, mainly in the lymph nodes. However, PD-1 suppressor T cells at a late stage in the immune response, mainly located in peripheral tissues. Therefore, CTLA-4 inhibitors tend to have greater side effects than do PD-1 inhibitors. Consequently, the combination of CTLA-4 and PD-1 inhibitors might cause heightened lung toxicity more than either immunosuppressant would alone. Despite this, further research is required to uncover the molecular mechanism behind this clinical observation. Therefore, awareness of the characteristics of pneumonitis associated with ICI may ensure the proper use of ICI in clinical practice, as well as proper patient monitoring after ICI treatment.

Numerous potential limitations in this research must be considered when interpreting the results. First, the number of papers included in this meta-analysis was limited, especially concerning the results of the subgroup analysis. Second, we included data from studies that utilized different ICIs at different doses, which means we could have overlooked variations in OS and ORR outcomes between medications or due to dosage discrepancies. However, due to the small number of studies, we did not perform further analysis to investigate these issues at the time. Third, the OS data from the included studies were immature, necessitating a future update with definitive OS data.

## 5. Conclusions

In this review, despite being limited by the small number of studies, the use of PD-1/PD-L1 inhibitors as monotherapy did not seem to be an attractive alternative to chemotherapy alone in mUC patients of first-line treatment or PD-L1 status ≥ 5%, except for platinum-ineligible patients. However, second-line therapeutic options in mUC show an OS advantage in immunotherapy over chemotherapy. In addition, treatment with a PD-1/PD-L1 inhibitor plus chemotherapy showed significant improvements in OS, PFS, and ORR. Therefore, our results provide evidence of a balance between the safety and benefits of PD-1/PD-L1 inhibitors, based on reconstructed survival data. However, further studies are needed to clarify the long-term efficacy and safety of PD-1/PD-L1 inhibitors and to determine which patients will benefit most from treatment with these agents.

## Figures and Tables

**Table 1:** Baseline characteristics of studies included in the meta-analysis.

Study	NCT No.	Phase	Line of Therapy	Treatment Arm	Number of Patients (Female)	Mean Age (Year)	Number of PD-L1 (IC2/3)	PFS, HR (95% CI)	OS, HR (95% CI)	ORR, No./Total No. (%)
**PD-L1 inhibitor**										
Galsky 2020 [28]	NCT02807636 (IMvigor130)	III	First	Atezolizumab plus chemotherapy	451 (113)	69	NR	0.82 (0.70–0.96)	0.83 (0.69–1.00)	212/447 (47.4)
				Atezolizumab	400 (102)	67	88	NR	1.02 (0.83–1.24)	82/359 (22.8)
				Platinum-based chemotherapy	362 (82)	67	85			174/397 (43.8)
Powles 2018 [29]	NCT02302807 (IMvigor211)	III	Second	Atezolizumab	467 (110)	67	116	NR	0.85 (0.73–0.99)	52/467 (11.1)
				Vinflunine or Taxanes	464 (103)	67	118			52/464 (11.2)
Powles 2020 [30]	NCT02516241 (DANUBE)	III	First	Durvalumab	346 (97)	67	209	1.10 (0.93–1.30)	0.99 (0.83–1.17)	89/346 (25.7)
				Platinum-based chemotherapy	344 (70)	68	207			169/344 (49.1)
**PD-1 inhibitor**										
Bellmunt 2017 [31]	NCT02256436 (KEYNOTE-045)	III	Second	Pembrolizumab	270 (70)	67	74	0.98 (0.81–1.19)	0.73 (0.59–0.91)	57/270 (19.3)
				Vinflunine or Taxanes	272 (70)	65	90			31/272 (11.4)
Powles 2021 [32]	NCT02853305 (KEYNOTE-361)	III	First	Pembrolizumab plus chemotherapy	351 (79)	69	NR	0.78 (0.65–0.93)	0.86 (0.72–1.02)	192/351 (54.7)
				Pembrolizumab	307 (79)	68	160	1.32 (1.09–1.58)	0.92 (0.77–1.11)	93/307 (30.3)
				Platinum-based chemotherapy	352 (90)	69	158			158/352 (44.9)

CI, confidence interval; HR, hazard ratios; NR, not reported; ORR, objective response rate; OS, overall survival; PD-1, programmed death-1; PFS, progression-free survival; PD-L1, programmed death-ligand 1.

**Table 2:** The quality assessment of 10 randomized controlled trials was included.

Study	Generation of the Allocation Sequence	Concealment of the Allocation Sequence	Blinding of Participants and Researchers	Blinding of Outcome Assessment	Incomplete Outcome Data	Selective Reporting	Other Bias
Galsky 2020 [28]	Low	Low	High	High	Low	Low	Unclear
Powles 2018 [29]	Low	Low	Low	Low	Low	Low	Unclear
Powles 2020 [30]	Low	Low	Low	Low	Low	Low	Unclear
Bellmunt 2017 [31]	Low	Unclear	High	Low	Low	Low	Unclear
Powles 2021 [32]	Low	Low	Low	Low	Low	Low	Unclear

**Table 3:** Risk of grade ≥ 3 adverse events in patients with bladder cancer treated with immune checkpoint inhibitors.

Grade ≥ 3 Adverse Events	No. of Trials	Events/Total Treatment Group	Events/Total Control Group	RR (95% CI)	*p*-Value
Hematologic	5	53/5361	675/5099	0.05 (0.02–0.10)	<0.001
Anemia	5	48/1825	270/1743	0.12 (0.06–0.27)	0.007
Leukopenia	2	1/611	25/568	0.06 (0.01–0.29)	0.69
Neutropenia	5	2/1825	274/1743	0.02 (0.01–0.05)	0.49
Thrombocytopenia	2	2/1100	106/1045	0.03 (0.01–0.09)	0.54
Non-hematologic	5	64/9954	187/9529	0.41 (0.28–0.59)	0.13
Asthenia	4	22/1559	44/1488	0.49 (0.29–0.83)	0.65
Constipation	2	0/725	28/698	0.03 (0.00–0.26)	0.66
Decreased appetite	4	15/1366	13/1300	1.16(0.54–2.47)	0.81
Fatigue	5	16/1825	53/1743	0.30 (0.17–0.54)	0.79
Nausea	3	4/1366	23/1300	0.25 (0.09–0.66)	0.64
Vomiting	2	2/647	15/655	0.18 (0.05–0.69)	0.54
Pruritus	4	1/1366	1/1300	0.93 (0.10–8.99)	0.35
Rash	3	4/1100	10/1045	0.97 (0.04–21.22)	0.04

CI, confidence interval; RR, risk ratio.

**Table 4:** Subgroup meta-analysis using various factors.

Variable	PFS (95% CI)	*p*	OS (95% CI)	*p*	ORR (95% CI)	*p*
ICI plus CT	0.80 (0.71–0.90)	<0.001	0.85 (0.74–0.96)	0.01	1.30 (1.02–1.66)	0.03
Line of therapy	1.12 (0.95–1.32)	0.16	0.90 (0.81–1.00)	0.05	0.67 (0.38–1.19)	0.17
First-line	1.20 (1.00–1.43)	0.05	0.97 (0.87–1.08)	0.60	0.42 (0.33–0.53)	<0.001
Second-line	0.98 (0.81–1.32)	0.84	0.82 (0.69–0.93)	0.003	1.42 (0.69–2.94)	0.3
PD-L1 IC2/3 tumors	1.11 (0.96–1.29)	0.15	0.84 (0.70–1.00)	0.04	0.85 (0.48–1.50)	0.58
First-line	1.21 (1.02–1.43)	0.39	0.90 (0.76–1.07)	0.34	0.54 (0.39–0.76)	<0.001
Second-line	0.96 (0.76–1.22)	0.61	0.72 (0.48–1.09)	0.12	1.92 (0.55–6.68)	0.31

CI, confidence interval; CT, chemotherapy; ICI, immune checkpoint inhibitors; ORR, objective response rate; OS, overall survival; PD-L1, programmed death-ligand 1; PFS, progression-free survival.
